# Letter to the Editor: Regarding “The Role of Obesity in Pediatric Orthopedics”

**DOI:** 10.5435/JAAOSGlobal-D-20-00011

**Published:** 2020-02-19

**Authors:** Andreas Rehm, Azeem Thahir

**Affiliations:** Cambridge, United Kingdom

*To the Editor:* We read with interest the recent publication by Nowicki et al, “The Role of Obesity in Pediatric Orthopedics.” The authors reported in their review article that obesity in the pediatric cohort alters bone metabolism, increasing the risk of fracture. It is stated that obesity leads to a lower ratio of overall bone mass when compared with overall patient weight, referencing two review articles by Bialo and Gordon^1^ and Lazar-Antman and Leet^2^ of which neither article supports this statement. The former reports “The definitive effect of pediatric obesity on bone mass remains controversial,” and the latter reports “In children, the data are inconclusive for correlations between bone mineral density and fracture…” and “The literature is also unclear as to how bone mineral density is affected by childhood obesity.”

Nowicki et al further discuss that obese children have poorer balance and generate more force than normal-weight children with comparable mechanism of injury, contributing to an increased fracture risk in young childhood. Li et al^3^ evaluated data from the KID database and identified that from 1997 to 2012, only 1.08% of 202,286 children who presented with an upper- and/or lower-limb fracture in the United States were obese. Therefore, it was 16 times less likely for an obese child to sustain a fracture and 41 times less likely to sustain a distal humerus fracture compared with a nonobese child based on an estimated 17% of children being obese in the United States (https://www.cdc.gov/healthy schools/obesity/facts.htm). Proximal femoral fractures had by far the highest rate of obese patients (8.3%) which is still only half that of the cohort obesity rate.

Seeley et al^4^ reviewed the medical records (but not the radiographs) of 354 children with a supracondylar humerus fracture to evaluate whether there was an association between fracture complexity and obesity. Of 68 obese patients, 57 had a fracture which was defined as complex, but only one of these was an intraarticular fracture. Nowicki et al stated that obesity influences early epiphyseal plate closure, leading to more adult fracture patterns in younger children without there being any radiological study to support this. To support their statement, the authors linked the article by Seeley et al with an image of a radiograph of a child allegedly aged 10 years 9 months old from a different source showing a distal adult-type intraarticular humerus fracture with closed growth plates. This is misleading since it gives the impression that Seeley et al^4^ had identified that obese children present with a higher rate of complex intra-articular adult fracture patterns because of early fusion of growth plates which they did not.

The fact that obese children sustain by far less fractures compared with nonobese children opposes Nowicki et al's discussion of the effects of obesity on the growing skeleton and on pediatric trauma.
